# The Effects of Different Doses of Ketamine on Quality of Normal Ejaculated Sperm

**Published:** 2014-07-08

**Authors:** Forouzan Absalan, Alireza Ghannadi, Abdollah Zabihi

**Affiliations:** 1 Department of Anatomical Sciences, Medical Faculty, Jundishapur University of Medical Science, Ahvaz, Iran; 2 Shiraz- Human Assisted Reproductive Center, Shiraz, Iran

**Keywords:** Sperm, Membrane Integrity, DNA Fragmentation, Ketamine

## Abstract

**Background:**

Ketamine, an injectable anesthetic in human and animal medicine, is also a recreational drug used by young adults. The aim of this study is to evaluate the effects of ketamine on membrane integrity, DNA fragmentation and sperm parameters in humans.

**Materials and Methods:**

This prospective study was conducted on 40 males with normal semen samples over one month (August 2012). Subjects were randomly allocated to four groups (Control and case I, II and III) whose semen samples were adjusted to different concentrations of ketamine (1, 3, 5 µL) for one hour. Sperm analysis was performed for routine parameters, motility and morphology. Evaluation of membrane integrity and DNA fragmentation was done by eosin-Y staining and the sperm chromatin dispersion (SCD) test, respectively. The results were analyzed by ANOVA and Tukey’s tests. P≤0.05 was considered statistically significant.

**Results:**

Total sperm motility in all case groups were significantly lower compared with the control group. In case group III, progressive motility showed significant difference with case group II. After addition of ketamine, sperm had evidence of coiled tails in all case groups compared to the control group however this observation was not significant. Evaluation of membrane integrity showed the rate of necrospermia increased in all case groups. However, ketamine only significantly affected membrane integrity in case group III. SCD staining showed that in the control group nucleoids with medium halos (63.44 ± 1.2) were significantly different compared to the case groups I (15.44 ± 0.45), II (9.05±1.16) and III (10.55 ± 1.14), respectively. Between case groups, nucleoids with large and medium halos showed significant differences in case groups II and III compared with case group I. Nucleoids with medium halos were significantly different between case groups II and III.

**Conclusion:**

Ketamine, through its effect on membrane integrity and DNA fragmentation, decreased sperm viability and caused abnormal sperm parameters in progressive motility.

## Introduction

Ketamine, a non-competitive antagonist at the glutamatergic N-methyl-D-aspartate (NMDA) receptor is currently used in human and animal medicine as an injectable anesthetic. Ketamine is also a controlled substance, illegally used as a recreational drug (1). In the last five years the percentage of ketamine users has rapidly grown. The reason for prevalent use of ketamine is that people believe it will not lead to physical dependence (2). Ketamine is absorbable via intravenous, intramuscular, oral, and topical routes due to both its water and lipid solubility. It is very short-acting, with hallucinatory effects that last 60 minutes when insufflated or injected and up to two hours when ingested orally, the total experience lasting no more than a couple of hours (3). At subanesthetic doses, ketamine produces a dissociative state, characterized by a sense of detachment from one’s physical body and the external world which is known as depersonalization and derealization (4). At sufficiently high doses, users may experience what is called the "K-hole", a state of dissociation whose effects are thought to mimic the phenomenology of schizophrenia (5). It has been shown that recreational ketamine ingestion has been associated with measurable concentrations of the drug in the blood, urine, brain tissue, spleen, liver and kidneys, and it is excreted in urine and bile (6). Acute dysfunction of visual convergence, poor body coordination and balance, and an increased pulse rate have been observed immediately after ketamine use (7).

Some of the effects of ketamine on the nervous system have been identified. Earlier studies have reported ketamine use to have a significant detrimental effect on memory function (8). As an acute effect, ketamine mainly activates the prefrontal cortex and the limbic structures (9). It can inhibit reuptake of serotonin, dopamine and norepinephrine, however the mechanism underlying this action is not entirely clear (10). Besides the toxicity to the nervous system, ketamine can affect the lower urinary tract, causing nocturia, dysuria, increased urinary frequency and sometimes hematuria (11, 12).

Based on ketamine effects on the urinary system, we hypothesize that the genital system may also be affected; therefore we have investigated the effects of different doses of ketamine on quality of normal human ejaculated sperm DNA and membrane integrity. Recently, failure of the conventional semen parameters to predict fertilization show that hidden anomalies lie at the sperm membrane level or at the chromatin level. Plasma membrane function activity is one of the most important aspects of sperm biology that involves metabolic exchanges with the surrounding medium which play an important role in several events during fertilization, capacitation, acrosomal reaction and sperm-oocyte fusion (13).

Investigation of membrane integrity seems to offer more information about sperm fertility potential than sperm parameters and probably there is relationship between sperm membrane integrity and sperm parameters (14, 15). We have used a rapid, simple test for this evaluation, the eosin-Y staining test (16, 17). In addition, the integrity of sperm DNA is being recognized as a new parameter of semen quality and a potential fertility predictor. In the present study we have analyzed DNA integrity of sperm by the sperm chromatin dispersion (SCD) test based on the principle that sperm with fragmented DNA fail to produce the characteristic halo of dispersed DNA loop observed in sperm with non-fragmented DNA following acid denaturation and removal of nuclear proteins (18).

## Materials and Methods

### Patient selection

In this prospective study, 40 males were enrolled at Shiraz-Human Assisted Reproductive Center during one month (August 2012). The semen samples were collected by masturbation after 2-5 days of sexual abstinence. After complete liquefaction, the semen were mixed with 2 or 3 Equal of Hams F-10 medium supplemented with 10% human serum albumin and then washed twice by centrifugation at 2000 rpm for 10 minutes until sperm separated from the semen. After obtaining the normal spermogram, normal samples were selected according to World Health Organization guidelines for the next survey. In the next step, 20 million motile sperm in 1 mL of medium were adjusted to different concentrations of ketamine (1, 3, 5 μL) for one hour. These doses were selected after ketamine was added to the medium and the viability rate of sperm was observed. Sperm analysis was performed for routine parameters, motility and morphology. Evaluation of DNA and membrane integrity was done by the SCD test and eosin-Y staining, respectively.

### Sperm chromatin dispersion (SCD) test

Aliquots of 0.2 mL of fresh semen samples were diluted in medium to obtain sperm concentrations that ranged from 5 to 10 million/mL. The suspensions were mixed with 1% low-melting-point aqueous agarose to obtain a 0.7% final agarose concentration at 37˚C. Aliquots of 50 μL of the mixture were pipetted onto slides which were carefully covered with coverslips. The slides were immediately immersed horizontally in a tray with freshly prepared acid denaturation solution (0.08 N HCl) for 7 minutes. A glass slide precoated with 0.65% standard agarose dried at 80˚C, covered with a coverslip (24 x 60 mm), and left to solidify at 4˚C for 4 minutes. The agarose matrix allows keeping the sample at 22˚C is resulted into generation of restricted single- stranded DNA (ss DNA) from DNA breaks. The denaturation was then stopped and proteins were removed by transferring the slides to a tray with neutralizing and lysing solution (0.4 M Tris, 0.8 M DTT, 1% SDS, 2 M NaCl, and 0.05 M Triplex) for 25 minutes at room temperature. Removal of nuclear proteins results in nucleoids with a central core and a peripheral halo of dispersed DNA loop. Slides were thoroughly washed twice in water for 5 minutes, dehydrated in sequential 70%, 90% and 100% ethanol baths (2 minutes each), and air dried. Cells were finally stained with Wright and PBS (1:1) for 10 minutes. After they were air dried, the degree of DNA dispersion was assessed by bright field microscopy. A minimum of 200 spermatozoa were evaluated by two different observers (19).

### Eosin-Y staining

This staining was performed by mixing 10 ml of sperm sample with 10 ml of dye (0.5% w/v; Merck Chemical CO., Germany) on a microscope slide which was then covered with a coverslip. A total of 200 sperm cells were counted within a few minutes after the addition of the dye (16-18). Evaluation of live (unstained) and dead (red stained) spermatozoa was undertaken by light microscopy observation at ×400 magnification.

In this study, all human research was approved by Shiraz-Human Assisted Reproductive Center (Shiraz, Iran) with the help of Ahvaz Jundishapur Medical University.

### Statistical analysis

The results were analyzed by ANOVA and repeated analysis for repeated measurement with p≤0.05 considered statistically significant. The mean and standard deviation (SD) was also calculated for each value.

## Results

There were control and three case groups of 10 patients per group. The results of sperm parameters at different doses of ketamine are reported in table 1. Total sperm progressive motility (fast and slow) in all case groups decreased significantly compared with the control group (p≤0.05). In case group III, there was a significant difference in progressive compared with case group II (p≤0.05). The mean percentage of sperm morphology decreased slightly in all case groups compared with the control group, but it was not significant.

**Fig 1:**
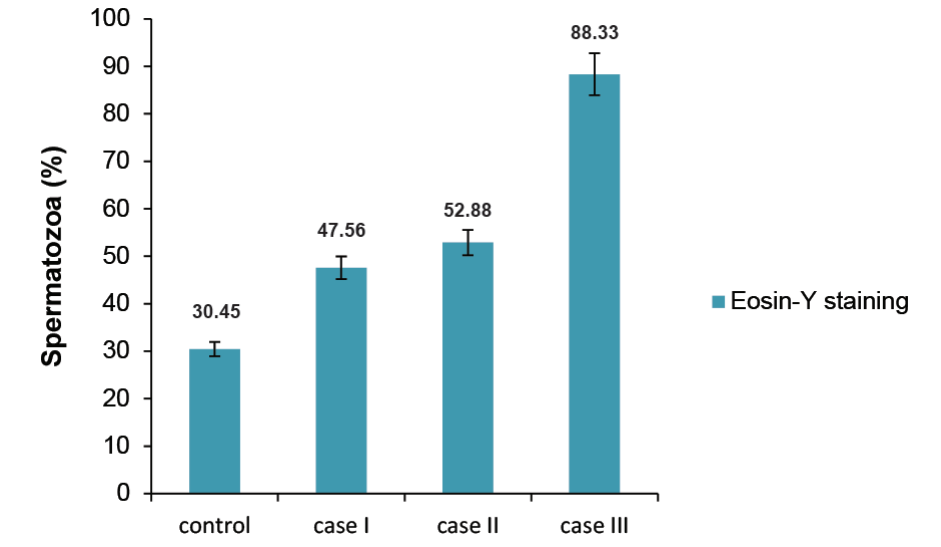
Comparison of sperm membrane integrity, evaluated by eosin-Y staining between control and case groups (mean ± SD). a; Difference between case group III and control group.

We evaluated membrane integrity using Eosin-Y staining and observed an increased rate of necrospermia in the case groups compared to the control group. However, this result was significant only in case group III (p≤0.05; Fig 1).

**Table 1:** The difference in human sperm parameters between control and case groups (mean ± SD)


Sperm parameters	Control group	Case group I	Case group II	Case group III

**Progressive motility (a+ b%)**	70.17 ± 1.12	47.48 ± 1.33 *	39.35 ± 1.4 *	29.95 ± 1.2 */**
**Morphologic alteration (%)**	71.38 ± 1.26	68.55 ± 1.33	54.44 ± 1.4	48.06 ± 1.27


*; Significant difference between case group I and control group and **; Significant difference between case group II and control group.

**Fig 2:**
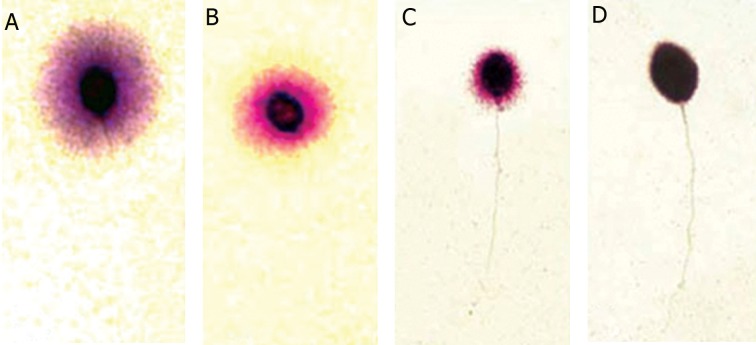
Nucleoides from human sperm cells obtained with the sperm chromatin dispersion (SCD) test. Nucleoides with large halos of DNA dispersion A. medium halos, B. small halos, C. no halos and D. degenerated.

Determination of sperm DNA fragmentation by the SCD test using conventional bright-field microscopy showed the presence of four SCD patterns as seen in figure 2: 1. sperm cells with large halos whose halo width was similar or higher than the minor diameter of the core; 2. sperm cells with medium size halos whose halo size is between those with large and small halos; 3. sperm cells with small size halo where the halo width is similar or smaller than one third of the minor diameter of the core; and 4. sperm cells without halos.

Table 2 showed a substantial difference in the percentage of positive SCD stained spermatozoa. In the control group nucleoids with medium halo (63.44 ± 1.2) showed a significant difference with all case groups I (15.44 ± 0.45), II (9.05 ± 1.16) and III (10.55 ± 1.14), respectively. Between case groups, nucleoids with large and medium halos showed significant differences in case group II and III compared with case group I. Nucleoids with medium halo had a significant difference between case groups II and III (p≤0.05).

**Table 2:** Sperm chromatin dispersion (SCD) data (mean ± standard error of mean) from semen samples between case groups and control group


Sperm sample	Large halo (%)	Medium halo (%)	Small halo (%)	No halo (%)

**Control group (n=10)**	28.11 ± 1.22	63.44 ± 1.2	7.88 ± 1.33	10.33 ± 1.1
**Case group I (n=10)**	21.88 ± 1.32	15.44 ± 0.45 ^a^	8.11 ± 1.33	10.55 ± 0.73
**Case group II (n=10)**	7.8 ± 1.33 ^a, b^	9.5 ± 1.16 ^a, b^	11.11 ± 1.25	10.66 ± 1.33
**Case group III (n=10)**	10.33 ± 1.33 ^a, b^	10.55 ± 1.14 ^a, b, c^	10.66 ± 1.2	12.66 ± 1.33


a; Significant difference between case group I and control group, b; Significant difference between case group II and control group and c; Significant difference between case group III and control group.

## Discussion

Ketamine is a dissociative anesthetic developed in 1963 to replace PCP and presently used in human anesthesia and veterinary medicine. Most ketamine sold on the street has been obtained from veterinarians’ offices. Ketamine is a slightly acidic solution administered through injection whose chemical name is (±)-2-(o-chlorophenyl)-2-(methylamino) cyclohexanone hydrochloride (20). Although it is manufactured as an injectable liquid, in illicit use ketamine is generally evaporated to form a powder and is either snorted or swallowed. Ketamine is odorless and tasteless, so it can be mixed with beverages without being noticed and it induces amnesia. Because of these properties, the drug is sometimes given to unsuspecting victims and used in the commission of sexual assaults referred to as "drug rape" (21, 22). Ketamine goes by various names on the street. It has been known to be called K, Special K, Cat valium, or vitamin K. Ketamine is abused in both the liquid and solid form. The liquid form is usually injected intravenously and is the most dangerous way to consume. The solid form is smoked, snorted or swallowed as a pill and it is known to be added to drinks (23, 24).

In the 1990s ketamine abuse, as a new type of drug abuse, appeared in areas of Southeastern China and in high-populated cities and spread quickly into mainland China (25). According to the 2010 drug abuse monitoring report by the State Food and Drug Administration (SFDA), the proportion of new type drug users has reached 28%. Most new type drug users are reported to be young, unemployed individuals in their fertility years (under 35 years of age). Over 80% have used Amphetamine-type stimulants (ATS) and the remainder used other drugs mostly for recreational purposes (26). It is likely that new type drugs will become the most extensive toxic abuse drugs in use in the 21st century instead of the traditional illegal psychoactive drug substances such as heroin and cocaine (26).

Until now researchers have mainly studied the neurological and urinary tract effects of ketamine. For the first time, Tan et al. (27) in 2011, have evaluated the effects of injection ketamine on sperm motility in mice. They found decreased sperm motility after ketamine treatment and suggested that long term ketamine treatment might affect the sperm environment leading to a decline in sperm motility that can affect fertility. In this manner our investigation about the effects of ketamine on quality of normal ejaculated sperms, DNA fragmentation and membrane integrity is not done. For the purposes of this research, we have added ketamine drug to semen samples.

It has been suggested that sperm concentration, motility and normality are three important parameters to evaluate infertility (28). We have found noticeable impact on parameters of human sperm following ketamine adjustment, which included decreased total motility, progressive motility and increased non-progressive motility, but it probably produced slight changes in sperm morphology (coiled tail). Though the mechanism of ketamine effects on sperm parameters is unknown, numerous clinical models support the relationship between semen parameters and increase in reactive oxygen species (ROS).

Oxidative stress (OS) represents an imbalance between the productions of ROS or free radicals and the available antioxidant system (29). Pasqualotto et al. (28) has shown that increase in ROS in semen affected semen parameters. Researchers have shown that antioxidant levels in the blood correlated with sperm counts and motility (30, 31).

On the other hand, ROS has been shown to correlate with reduced male fertility by causing peroxidative damage to the sperm plasma membrane (32). OS leads to lipid peroxidation of sperm membranes rich in unsaturated fatty acids and it has been shown that sperm motility is very closely correlated with sperm membrane integrity (33, 34). The inverse correlation between lipid peroxidation and sperm motility has also been shown by Giwercman et al. (35). Our findings agreed with the above mentioned research and showed a dose-dependent effect of ketamine on membrane integrity. The current studies on membrane integrity subsidiary shows decrease of it by ketamine may result into ROS function.

It has been shown that OS is a major case of infertility and is intimately related to DNA fragmentation (35, 36). Both testicular and extratesticular factors contribute to the final load of sperm DNA damage in ejaculated sperm. It has been reported that ROS might cause several forms of sperm DNA damage such as single and double strand DNA breaks (37). Superoxide and hydrogen radicals are mutagenic species and can cause chromosomal damage (38). In addition, a relationship between sperm motility and DNA damage was shown as a result of the adverse effects of OS (39).

Our findings of the chromatin dispersion pattern according to the SCD test after ketamine dosing have shown that abnormalities in sperm parameters reflected DNA structure.

## Conclusion

Our results demonstrate that ketamine administration significantly decreases sperm motility, viability and normal chromatin dispersion. These results may disturb male reproductive function.
